# Characterization of Long Non-coding RNAs Modified by m^6^A RNA Methylation in Skeletal Myogenesis

**DOI:** 10.3389/fcell.2021.762669

**Published:** 2021-10-13

**Authors:** Shu-Juan Xie, Shuang Tao, Li-Ting Diao, Pan-Long Li, Wei-Cai Chen, Zhi-Gang Zhou, Yan-Xia Hu, Ya-Rui Hou, Hang Lei, Wan-Yi Xu, Wen-Jie Chen, Yan-Wen Peng, Qi Zhang, Zhen-Dong Xiao

**Affiliations:** ^1^Vaccine Research Institute of Sun Yat-sen University, The Third Affiliated Hospital of Sun Yat-sen University, Guangzhou, China; ^2^Biotherapy Center, The Third Affiliated Hospital of Sun Yat-sen University, Guangzhou, China; ^3^Department of Orthopedics, First Affiliated Hospital, Jinan University, Guangzhou, China

**Keywords:** m^6^A, lncRNAs, Brip1os, METTL3, skeletal muscle development

## Abstract

Proper development of mammalian skeletal muscle relies on precise gene expression regulation. Our previous studies revealed that muscle development is regulated by both mRNA and long non-coding RNAs (lncRNAs). Accumulating evidence has demonstrated that N^6^-methyladenosine (m^6^A) plays important roles in various biological processes, making it essential to profile m^6^A modification on a transcriptome-wide scale in developing muscle. Patterns of m^6^A methylation in lncRNAs in developing muscle have not been uncovered. Here, we reveal differentially expressed lncRNAs and report temporal m^6^A methylation patterns in lncRNAs expressed in mouse myoblasts and myotubes by RNA-seq and methylated RNA immunoprecipitation (MeRIP) sequencing. Many lncRNAs exhibit temporal differential expression, and m^6^A-lncRNAs harbor the consensus m^6^A motif “DRACH” along lncRNA transcripts. Interestingly, we found that m^6^A methylation levels of lncRNAs are positively correlated with the transcript abundance of lncRNAs. Overexpression or knockdown of m^6^A methyltransferase METTL3 alters the expression levels of these lncRNAs. Furthermore, we highlight that the function of m^6^A genic lncRNAs might correlate to their nearby mRNAs. Our work reveals a fundamental expression reference of m^6^A-mediated epitranscriptomic modifications in lncRNAs that are temporally expressed in developing muscle.

## Introduction

Skeletal muscle plays critical roles in the regulation of wider metabolism as well as driving locomotion (Cong et al., 2020). Myogenesis, the development of muscle, is a complex biological process regulated by multiple transcription factors and specific signaling pathways (Bryson-Richardson and Currie, 2008; Bentzinger et al., 2012). Our previous studies showed that non-coding RNAs, including miRNAs and lncRNAs (long non-coding RNAs), play essential roles in skeletal muscle development (Xie et al., 2018; Liu et al., 2021; Tan et al., 2021). LncRNAs are a class of non-coding RNAs greater than 200 nucleotides in length with limited or no protein-coding capacity that possess complex spatial structures and diverse functions (Chen and Carmichael, 2010; Xie et al., 2021a). Numerous studies have shown that lncRNAs play a significant role in biological functions, such as epigenetic modification, mRNA transcription, splicing, stability and translation (Lan et al., 2021). Functionally, lncRNAs can either act in *cis* by regulating the expression of neighboring genes or in *trans* by regulating the expression of distant genes (Ulitsky and Bartel, 2013). Increasing studies have shown that lncRNAs participate in myogenesis. For example, H19, one of the earliest known imprinted lncRNAs, is strongly repressed after birth in all mouse tissues, but it remains expressed in the skeletal muscle and heart in adults (Milligan et al., 2000), controlling reactivation of the imprinted gene network and alleviating muscular dystrophy (Borensztein et al., 2013; Martinet et al., 2016; Zhang Y. et al., 2020). LncRNA MALAT1 interacts with miRNAs or mRNAs to regulate skeletal muscle maintenance (Yong et al., 2020; Liu et al., 2021). Additionally, several lncRNAs have been reported to shape muscle (Ro et al., 2018; Sweta et al., 2019; Martone et al., 2020), including linc-MD1 (Cesana et al., 2011), lincYY1 (Lu et al., 2013; Zhou et al., 2015), lncRNA Dum (Wang et al., 2015), linc-RAM (Yu et al., 2017; Zhao et al., 2018), muscle-specific lncR-Irm (Sui et al., 2019), lncR-Myoparr (Hitachi et al., 2019), lnc-MyoD (Gong et al., 2015; Lim et al., 2020), and lncMGPF (Lv et al., 2020).

RNA chemical modifications in coding and non-coding RNAs can regulate gene expression without changing the sequence of the RNA molecules via a process referred as “epitranscriptomics” (Helm and Motorin, 2017; Roundtree et al., 2017; Fazi and Fatica, 2019). Greater than 150 RNA modifications have been identified as posttranscriptional regulatory marks in multiple RNA species, including mRNAs, tRNAs, rRNAs, small non-coding RNAs, and lncRNAs (Yang Y. et al., 2018). Among these modifications, N^6^-methyladenosine (m^6^A) is the most common modification in mammalian mRNAs and lncRNAs (Pan, 2013). The effectors in m^6^A pathways include “writers” and “erasers” that install and remove the methylation, respectively, and “readers” that recognize it (Fu et al., 2014; Meyer and Jaffrey, 2017; Shi et al., 2019). The m^6^A writer machinery is a methyltransferase complex composed of multiple subunits with a stable core complex formed between methyltransferase-like 3 (METTL3) and methyltransferase-like 14 (METTL14) (Ping et al., 2014). Benefitting from deep sequencing, m^6^A patterns have been demonstrated to occur in a cell type- and cell state-dependent manner, and the landscape of the m^6^A methylome has been identified in human and mouse tissues (Liu et al., 2020; Zhang H. et al., 2020).

Given the important function of m^6^A modification in gene expression, emerging evidence has revealed the critical role of m^6^A in skeletal muscle regulation (Li et al., 2021). Our recent study elaborated the dynamic m^6^A methylation of mRNA during skeletal muscle differentiation and revealed the role of METTL3/14-m^6^A-MNK2-ERK signaling axis in skeletal muscle differentiation and regeneration (Xie et al., 2021b). Besides, another work of us confirmed that the m^6^A key methyltransferase METTL3 is involved in the biogenesis of muscle-specific miRNAs (Diao et al., 2021a). This finding is consistent with a previous report indicating that METTL3 is sufficient to enhance miRNA maturation in a global and non-cell-type specific manner (Alarcón et al., 2015). Other studies focused on the modification of mRNA by m^6^A methylation, which is involved in muscle formation, maintaining muscle homeostasis, and musculoskeletal disorders (Zhang W. et al., 2020). Recent studies revealed that METTL3-mediated m^6^A methylation is essential for muscle stem cell self-renewal (Lin et al., 2020), muscle regeneration (Liang et al., 2021), and muscle stem cell/myoblast state transitions (Gheller et al., 2020). Interestingly, myogenic potential is maintained partly by the Mettl3-mediated stabilization of processed MyoD mRNA through m^6^A modification of the 5′ untranslated regions (UTR) during proliferative phases (Kudou et al., 2017), and depletion of m^6^A “eraser” FTO in myoblasts leads to impaired skeletal muscle development (Wang et al., 2017). These data suggested that m^6^A modification could mediate muscle progenitor cell proliferation and differentiation. It has been demonstrated that m^6^A lncRNA modification plays roles in different biological processes (Patil et al., 2016; Yang D. et al., 2018; Fazi and Fatica, 2019; Ma et al., 2019; He et al., 2020; Lan et al., 2021). However, little is known about the m^6^A methylation status of lncRNAs involved in developing skeletal muscle.

In this study, we characterized lncRNAs in mouse myoblasts and differentiated myotubes using RNA sequencing (RNA-seq) and further uncovered abundant m^6^A sites and specific m^6^A patterns in these lncRNAs using methylated RNA immunoprecipitation sequencing (MeRIP-seq). Our results reveal the temporal expression profile and m^6^A methylation status of lncRNAs during skeletal myogenesis. We found that the m^6^A methylation levels of lncRNAs were positively correlated with their transcriptional abundance. Our data will provide a fundamental reference for further study on the function of lncRNAs.

## Materials and Methods

### Cell Culture

The C2C12 mouse myoblast cell line and HEK-293T cells were purchased from the Cellular Library of the National Collection of Authenticated Cell Cultures (Shanghai, China). The cells were cultured in growth medium (GM)-Dulbecco’s modified Eagle’s medium (DMEM, Gibco) with 10% fetal bovine serum (FBS, Gibco), 100 U/ml penicillin, and 100 μg/ml streptomycin (1 × penicillin–streptomycin) at 37°C in a humidified chamber supplemented with 5% CO_2_. When C2C12 cells reached about 90% confluency, the GM was replaced with differentiation medium (DM)-DMEM containing 2% horse serum (HyClone).

### Stable Cell Generation

For METTL3 overexpression, cDNA of mouse METTL3 was cloned into the pKD-CMV-MCS-EF1-PURO (pKD) vector by Gibson Assembly, and pKD-GFP was used as a negative control. For METTL3 knockdown, the gRNAs downstream of the transcription start sites were used to guide the fusion of inactive Cas9 (dCas9) to the Krüppel-associated box (KRAB) repressor. To generate stable cells, lentiviruses were produced in HEK-293T cells by transfecting vectors together with psPAX.2 and pMD2.G. Lentiviruses were collected and filtered 48 h after transfection and then used to infect target cell lines. Stable cells were selected using the antibiotic puromycin.

All sequences of clone primers used in this study are listed in Supplementary Table 1.

### Gene Knockdown

To knock down lncRNAs, custom designed siRNAs targeting selected lncRNAs and control siRNAs were synthesized by Shanghai GenePharma Co., Ltd. C2C12 cells were seeded in 12-well plates and transfected with siRNAs using Lipofectamine 2000 (Invitrogen) after the cells reached 30–40% confluency, according to the manufacturer’s instructions. The transfected cells were maintained in growth medium for two days, and then cells were harvested for analysis.

All siRNA sequences used in this study are listed in Supplementary Table 2.

### m^6^A Dot Blot Assay

RNA dot blotting was performed as previously described with modifications (Chen et al., 2015). Cells were harvested carefully and purified using a Dynabeads mRNA direct kit (Invitrogen, 61012). To avoid RNA degradation, RNase-free tubes and RNase-free water were used. RNA samples were quantified, diluted and incubated at 95°C in a heat block for 3 min to disrupt secondary structures. The tubes were chilled on ice immediately after denaturation for 2 min. The RNA samples were dropped onto the membrane (Amersham Hybond-N ^+^, GE) and allowed to air dry for 5 min. Then, RNA was crosslinked to the membrane with UV light (2 autocrosslink, 150 mJ/cm^2^ UV Stratalinker, STRATAGENE). The membrane was washed in TBST (1TBS, 0.1% Tween-20), dyed in methylene blue (Sigma–Aldrich) as a quantitative control, and incubated in blocking buffer containing 5% non-fat dry milk in TBST for 2 h at room temperature. Then, the membrane was incubated with m^6^A antibody (1:1000, Synaptic Systems, 202-003) at 4°C overnight. The membrane was washed 3 times for 10 min each in TBST and then incubated with HRP-linked secondary anti-rabbit IgG antibody (1:5000, CST, 7074) for 1 h at room temperature. Signals were detected with WesternBright ECL HRP substrate (Advansta). The dots were quantified using ImageJ.

### Western Blot

Cells were lysed in ice-cold enhanced RIPA lysis buffer (Shanghai Wansheng Haotian Biological Technology) containing phosphatase inhibitor and protease inhibitor cocktail (Roche). Equivalent total protein extracts were separated by SDS–PAGE and transferred to nitrocellulose membranes (Merck Millipore). The membranes were blocked with 5% non-fat dry milk in TBST for 1 h at room temperature. The following antibodies were used in this study: anti-METTL3 (Proteintech, 15073-1-AP), anti-METTL14 (R&D, HPA038002), anti-MHC (R&D, MAB4470), and anti-GAPDH (CST, 2118). Immunoreactivities were determined using WesternBright ECL HRP substrate (Advansta).

### RNA Isolation and Quantitative RT-PCR Assay

Total RNA was extracted from cells with TRIzol reagent (Invitrogen, 15596018). First-strand cDNA for PCR analyses was synthesized with HiScript III RT SuperMix for qPCR (+ gDNA wiper) (Vazyme, R323-01), and quantitative real-time PCR was performed using ChamQ Universal SYBR qPCR Master Mix (Vazyme, Q711-02). The GAPDH gene served as an endogenous control. The qRT-PCR results were analyzed and presented as relative RNA levels of the CT (cycle threshold) values, which were then converted as fold change. The results are presented as the means ± SD.

All primers for qPCR are listed in Supplementary Table 1.

### RNA Library Construction and Sequencing

Total RNA was isolated and purified using TRIzol reagent (Invitrogen, 15596018) following the manufacturer’s procedure. The RNA amount and purity were quantified by a NanoDrop ND-1000 (NanoDrop). RNA integrity was assessed using the Agilent 2100 system with RIN number >7.0 and confirmed by electrophoresis with a denaturing agarose gel.

RNA-seq was performed by BGI Co., Ltd. Briefly, total RNA was used to purify the poly-A containing RNAs using poly-T oligo-attached magnetic beads. Following the purification, the remainder of the RNA was fragmented into small pieces using divalent cations under high temperature. Then, the cleaved RNA fragments were reverse transcribed to create the cDNA library according to the mRNA-Seq sample preparation kit protocol (Illumina). The average insert size for the paired-end libraries was 300 bp (±50 bp). Then, paired-end sequencing was performed on an Illumina NovaSeq^TM^ 6000 (BGI Co., Ltd, Shenzhen, China) following the vendor’s recommended protocol.

### MeRIP Sequencing

RNA extraction and quality control were performed as previously noted. Poly(A) RNA was purified from 50 μg of total RNA using Dynabeads Oligo (dT) 25-61005 (Thermo Fisher) and fragmented into 100-nucleotide-long oligonucleotides using Magnesium RNA Fragmentation Module (NEB). The cleaved RNA fragments were immunoprecipitated using an anti-m^6^A affinity purified antibody (Synaptic Systems, 202003). Then, the IP RNA was reverse transcribed to cDNA by SuperScript^TM^ II Reverse Transcriptase (Invitrogen, 1896649) followed by U-labeled second-stranded DNA synthesis. Then, after ligation with the adapter to the A-tailed fragmented DNA, the ligated products were amplified with PCR, and 2 × 150 bp paired-end sequencing (PE150) was performed on an Illumina NovaSeq^TM^ 6000 (LC-Bio Technology CO., Ltd., Hangzhou, China) following the vendor’s recommended protocol.

### m^6^A-IP-qPCR

m^6^A methylated RNA immunoprecipitation was performed as previously described (Diao et al., 2021a). Briefly, 200 μg of total RNA was incubated with 8 μg of m^6^A-specific antibody (Synaptic Systems, 202003) for 2 h at 4°C with gentle rotation. Then, 50 μl protein A/G magnetic beads were added and incubated for 2 h at 4°C with gentle rotation. Beads were pelleted at 2,500 rpm for 30 s. Then, the supernatant was removed, and the beads are resuspended in 500 μL RIP buffer. The process is repeated for a total of three RIP washes followed by one wash in PBS. Beads were incubated with DNase I for 30 min at 37°C and were then digested by Proteinase K for 2 h at 37°C with rotation. A MicroElute RNA Clean Up Kit (Omega) was used for RNA purification. Purified RNA was reverse transcribed and quantified by real-time RT-PCR.

### RNA-Seq Analysis

The sequencing adapters were removed from raw fastq data using cutadapt (Kechin et al., 2017) software, and then clean reads were mapped to the mouse reference genome (mm10) using HISAT2 (Kim et al., 2019). According to reference gene annotation (Ensembl release 102), the raw counts of each gene were calculated using featureCounts (Liao et al., 2014). Raw counts were further normalized as reads per kilobase of genome per million mapped reads (RPKM) using the fpkm function in the DESeq2 (Love et al., 2014) package. Differentially expressed genes were identified using DESeq2 (Love et al., 2014) with adjusted *P* ≤ 0.05. LncRNAs were selected for downstream analysis based on gene type.

### MeRIP-Seq Analysis

The sequencing adapters were removed from raw fastq data using cutadapt (Kechin et al., 2017) software, and then clean reads were mapped to the mouse reference genome (mm10) using HISAT2 (Kim et al., 2019). Unique mapped reads were selected using samtools (Li et al., 2009) with a mapping quality greater than 30. To visualize read coverage using IGV (Robinson et al., 2011), the bigwig format of mapped reads was generated using deeptools (Ramírez et al., 2014). Mapped reads of IP and input libraries were fed into the exomePeak (Meng et al., 2013) package for calling peaks and identifying distinct peaks, and statistical significance was defined as FDR ≤ 0.05. Called peaks were annotated by intersecting with gene architecture using bedtools (Quinlan and Hall, 2010) and custom Python script. Peaks located at lncRNAs were selected based on gene type for downstream analysis.

### Motif Identification and Peak Distribution Among lncRNA Bodies

Sequence motifs enriched in m^6^A peaks were identified by HOMER (Heinz et al., 2010) with “−len 5,6,7,8 −rna” and other default settings as parameters. An m^6^A metagene plot was plotted using the Guitar (Cui et al., 2016) package. To investigate the peak distribution of lncRNA exon elements, an in-house Python script was developed to calculate the numbers of peaks located at the first exon, internal exons and last exon. Pie charts were plotted using ggplot2 package.

### Relationship Analysis of lncRNA m^6^A Level and Expression Abundance

Significant differential m^6^A peaks were integrated with corresponding differential expression data regardless of significance. Based on change orientation, the lncRNAs with significant differential m^6^A peaks were classified into four groups: hypermethylated and upregulated, hypermethylated and downregulated, hypomethylated and upregulated, and hypomethylated and downregulated. A four-quadrant diagram was generated using the ggplot2 package. To estimate the relationship of lncRNA m^6^A level and expression abundance, Pearson correlation analysis was performed using R software (version 4.0.3).

### Neighbor Gene Analysis of Differentially Expressed lncRNAs

Ten neighboring mRNAs (five upstream and five downstream) of differentially expressed lncRNAs were obtained using an in-house Python script, and mRNAs with significantly different expression (adjusted *P* ≤ 0.05) were fed into the clusterProfiler (Yu et al., 2012) package for GO and KEGG analysis. Statistical significance was defined as *p* ≤ 0.05. The top 10 most significant terms are shown using dot plots. For genes associated with muscle development, their neighboring lncRNAs were also required to have significant m^6^A changes. Finally, a set of lncRNAs with expression and m^6^A alterations related to muscle development were identified.

### Visualization Analysis

A heatmap was drawn using the pheatmap package, and other charts that were not specified were drawn using the ggplot2 package.

## Results

### Dynamic Profile of lncRNAs in Myoblasts and Differentiated Myotubes

**FIGURE 1:**
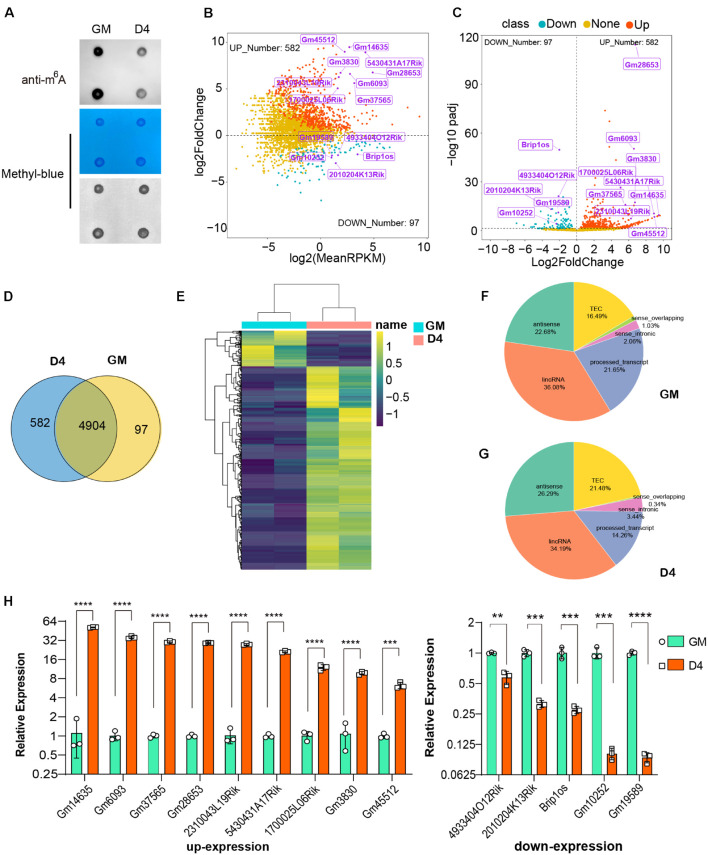
Dynamic profile of lncRNAs in myoblasts and differentiated myotube. **(A)** RNA dot blot assay of m^6^A methylation levels of C2C12 myoblasts in GM and D4. **(B)** MA plot shows the relationship of expression abundance and fold changes of lncRNAs in myoblasts (GM) and myotube (D4). log2 (MeanRPKM) represents gene expression values, log2Fold Change represents the fold change of lncRNAs at D4 compared to GM. Red dots represent 582 significantly up-regulated lncRNAs at D4 in relation to GM, adjusted *P* ≤ 0.05; Green dots, represent 97 significantly down-regulated lncRNAs at D4 in relation to GM, adjusted *P* ≤ 0.05; Yellow dots represent lncRNA without significantly differential expression, adjusted *P* > 0.05. Top differentially expressed lncRNAs were marked in purple. **(C)** The volcano plot shows significantly differentially expressed lncRNAs at GM and D4. Red dots represent 582 significantly up-regulated lncRNAs at D4, adjusted *P* ≤ 0.05; Green dots, represent 97 significantly down-regulated lncRNAs at D4, adjusted *P* ≤ 0.05; Yellow dots represent lncRNA without significantly differential expression, adjusted *P* > 0.05. Top differentially expressed lncRNAs were marked in purple. **(D)** The Venn diagram shows 97 and 582 significantly differentially expressed lncRNAs in GM and D4, and 4904 lncRNAs without significant difference between GM and D4. **(E)** The heatmap shows significantly differentially expressed lncRNAs in GM and D4. Yellow color represents up-regulation in D4, while green color represents down-regulation. Row represents genes, column represents samples, and each cell represents expression value. **(F)** Pie chart show the gene types of lncRNAs up-regulated in GM. **(G)** Pie chart show the gene types of lncRNAs up-regulated in D4. **(H)** Quantitative real-time reverse transcription PCR (qRT-PCR) validated top differently expressed lncRNAs in developing muscle cells. RPKM: Reads Per Kilobase per Million mapped reads. adjusted P: adjusted *p*-value; Data are presented as Mean ± SD; *p* value: **p < 0.01, ***p < 0.001, ****p < 0.0001.

We used mouse C2C12 myoblast cells to mimic skeletal muscle differentiation. C2C12 cells constantly proliferate in the presence of serum and begin to differentiate in the absence of serum. After differentiation for 4 days, obvious morphological changes were observed when myocytes fused to form multinucleated myotubes (Supplementary Figure 1A), as described in our previous reports (Xie et al., 2018; Tan et al., 2021). Next, RNA dot blotting was performed to investigate the dynamics of m^6^A RNA modification during myogenesis, and decreased global m^6^A levels were observed in myotubes that had been differentiated for 4 days (D4) compared to myoblasts (cells on growth medium, GM) (Figure 1A). To test whether this change was due to altered expression of m^6^A methyltransferases or demethylases, we profiled the core components of m^6^A methyltransferases METTL3, METTL14, and m^6^A demethylases FTO and ALKBH5 during C2C12 differentiation. Consistent with the decline in m^6^A levels, METTL3 and METTL14 protein expression was decreased on D4 and negatively correlated with the myogenic marker MHC (Supplementary Figure 1B). However, the demethylases FTO and ALKBH5 had opposite changes during C2C12 differentiation (Supplementary Figure 1C), and the upregulation of FTO is consistent with previous report (Wang et al., 2017). These data revealed that m^6^A and its core methyltransferase decreased during C2C12 differentiation. Such a dynamic change in m^6^A may contribute to the regulation of muscle genes in myoblasts and myotubes.

In addition to RNA modification, non-coding RNAs, especially long non-coding RNAs (lncRNAs), could represent robust gene expression regulators. To profile the dynamic changes in lncRNAs during C2C12 differentiation, RNA sequencing (RNA-seq) was performed to analyze the expression of lncRNAs in GM and D4. Using bioinformatic analysis, we detected 5,583 lncRNAs expressed in at least one sample. Of these, 679 differentially expressed lncRNAs were identified (adjusted *P* value ≤0.05, Supplementary Table 3), We found that these differentially expressed genes generally tended to exhibit increased expression (Figure 1B). Moreover, the number of upregulated genes was far greater than that of downregulated genes (Figures 1B,C,E). We identified 97 and 582 lncRNAs significantly differentially expressed in GM and D4, respectively (Figure 1D). The top differentially expressed lncRNAs are marked in purple and are listed in Table 1. Among these lncRNAs, the majority were lincRNAs with 36.08% in GM and 34.19% in D4. The second rank was antisense RNA in both GM and D4 samples (Figures 1F,G). To validate the RNA-seq results, we performed quantitative real-time reverse transcription PCR (qRT-PCR) for the top nine upregulated and top five downregulated lncRNAs. All tested lncRNAs showed significantly differential expression, which was consistent with the RNA-sequencing results (Figure 1H). Of note, the top highly expressed lncRNAs, including Gm14635, Gm28653, 2310043L19Rik and 5430431A17Rik, were also highly expressed in the limb. In particular, Gm28653, which is also known as linc-MD1, is a muscle-specific lncRNA that controls muscle differentiation (Cesana et al., 2011). In addition, lncRNA 1700025l06Rik has the same transcript locus as lincMyod, which is directly activated by MyoD and regulates skeletal muscle differentiation by blocking IMP2-mediated mRNA translation (Gong et al., 2015).

**TABLE 1:** Top differentially expressed lncRNAs in myoblasts and differentiated myotube.

Ensembl_ID	Gene_Name	Expression	log2FoldChange	meanfpkm	padj
ENSMUSG00000087591	Gm14635	Up	9.478458245	6.5058287	2.95E-10
ENSMUSG00000110071	Gm45512	Up	8.979513546	4.7889075	3.66E-09
ENSMUSG00000108322	5430431A17Rik	Up	8.917522207	18.467968	3.13E-11
ENSMUSG00000099906	Gm28653	Up	6.761660182	29.802335	4.47E-115
ENSMUSG00000099465	Gm3830	Up	6.719594193	3.649369	4.34E-18
ENSMUSG00000112963	Gm6093	Up	6.600023017	6.7770027	4.89E-51
ENSMUSG00000101746	2310043L19Rik	Up	6.259516887	3.2733601	3.43E-08
ENSMUSG00000104045	Gm37565	Up	5.616399462	8.984661	1.25E-16
ENSMUSG00000109962	1700025L06Rik	Up	5.062787459	3.0317699	3.23E-27
ENSMUSG00000085208	Brip1os	Down	–2.020791677	11.071526	2.06E-50
ENSMUSG00000097908	4933404O12Rik	Down	–2.110740583	3.1048072	6.83E-22
ENSMUSG00000100798	Gm19589	Down	–2.141593862	2.0967695	7.30E-12
ENSMUSG00000110350	Gm10252	Down	–2.36282624	2.0370561	6.34E-05
ENSMUSG00000063018	2010204K13Rik	Down	–2.962399389	2.6803628	6.84E-14

Meanwhile, we analyzed the mRNAs expression pattern of RNA-seq data, and identified 8,817 out of 16,190 differentially expressed mRNAs in D4 versus GM (adjusted *P* value ≤0.05, Supplementary Table 4). The number of upregulated coding genes was a little less than that of downregulated genes (Supplementary Figures 2A,B,D), of which 4483 and 4334 mRNAs significantly differentially expressed in GM and D4, respectively, and 7737 mRNA showed no significantly different expression in both group (Supplementary Figure 2C). The distinct difference in the number of genes with significant change between mRNAs and lncRNAs may due to the fact that mRNAs account for the majority of cellular RNA contents. Taken together, our RNA-seq analysis revealed the temporal expression of lncRNAs and mRNAs during myoblast differentiation.

### Features of lncRNA m^6^A Methylation in Undifferentiated and Differentiated Muscle

**FIGURE 2:**
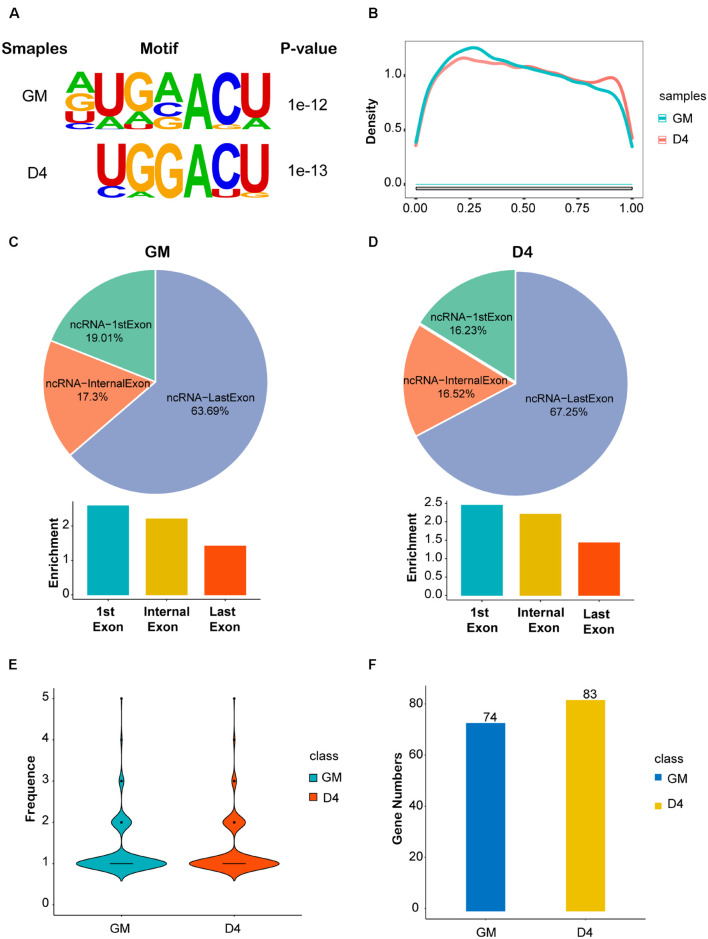
Features of lncRNA m^6^A methylation in undifferentiated and differentiated muscle. **(A)** The enriched consistent motif of m^6^A peaks in lncRNAs in GM and D4. **(B)** Metagene profiles of enrichment of all m^6^A peaks across lncRNAs transcriptome. **(C)** The top: pie charts represent the proportion of m^6^A peaks in the three regions of lncRNAs at GM. The bottom: histogram represents the relative enrichment of m^6^A peaks in the three regions of lncRNAs at GM. **(D)** The top: pie charts represent the proportion of m^6^A peaks in the three regions of lncRNAs at D4. The bottom: histogram represents the relative enrichment of m^6^A peaks in the three regions of lncRNAs at D4. **(E)** The frequence of m^6^A Peak Numbers in lncRNAs in GM and D4. **(F)** Bar plot shows the Numbers of m^6^A methylated lncRNAs in GM and D4. Blue represents 74 hyper-methylated lncRNAs in GM, while yellow represents 83 hyper-methylated lncRNAs in D4.

Given the great importance of m^6^A methylation and non-coding RNAs in skeletal muscle development (Martone et al., 2020) as well as the altered m^6^A modification and lncRNA expression profiles in myoblast differentiation, we conducted methylated RNA immunoprecipitation sequencing (MeRIP-seq) in GM and D4 samples to uncover the m^6^A methylation landscape of lncRNAs. We identified greater than 20 thousand unique peaks from each MeRIP-seq sequencing library (FDR ≤ 0.05, Supplementary Tables 5, 6). Among them, we found 1383 lncRNA m^6^A peaks in the GM sample and 1848 lncRNA m^6^A peaks in the D4 sample (FDR ≤ 0.05, Supplementary Tables 7, 8). And we also found 5122 significantly differently mRNA peaks in D4 versus GM, of which 2692 mRNA m^6^A peaks in the GM sample and 1442 mRNA m^6^A peaks in the D4 sample (FDR ≤ 0.05, Supplementary Table 9 and Supplementary Figure 3). To identify whether m^6^A peaks share common sequence elements, we analyzed m^6^A binding motifs using Homer. We detected a significantly enriched consensus motif DRACU within lncRNAs from myoblast GM and myotube D4 (Figure 2A). The motif matched the well-validated consensus m^6^A motif DRACH (where D = A, G or U; R = A or G; H = A, C or U) and was similar to a previous report in lincRNAs (Meyer and Jaffrey, 2017). We explored the distribution of peaks along the lncRNA gene body and found that its density gradually decreased from the transcription initiation site to the transcriptional termination site (Figure 2B), which differs from the coding genes (Liu et al., 2015).

We further analyzed the peak distribution of lncRNA exons. We found that m^6^A peaks were preferentially enriched in the last exon of lncRNAs expressed in GM and D4 samples. In total, 63.69% and 67.25% m^6^A peaks were identified in the last exon of lncRNAs expressed in GM and D4, respectively. We then analyzed the peak enrichment in each lncRNA. Interestingly, we found that m^6^A peaks are preferentially enriched in the first exon and internal exon, but not the last exons (Figures 2C,D). We then analyzed the numbers of m^6^A peaks within lncRNAs and identified a median value of 1.0 m^6^A peaks per lncRNA (Figure 2E), and no differences were noted between the GM and D4 data. Furthermore, we performed integrating analysis by coupling MeRIP-seq and RNA-seq data. We mapped m^6^A peaks to differentially expressed lncRNAs and found that 74 and 83 lncRNAs were significantly hypermethylated in myoblasts (GM) and myotubes (D4), respectively (Figure 2F). These data provide a fundamental reference for the m^6^A epitranscriptome for further study.

### Differentially m^6^A-Modified lncRNAs in Undifferentiated and Differentiated Muscle

**FIGURE 3:**
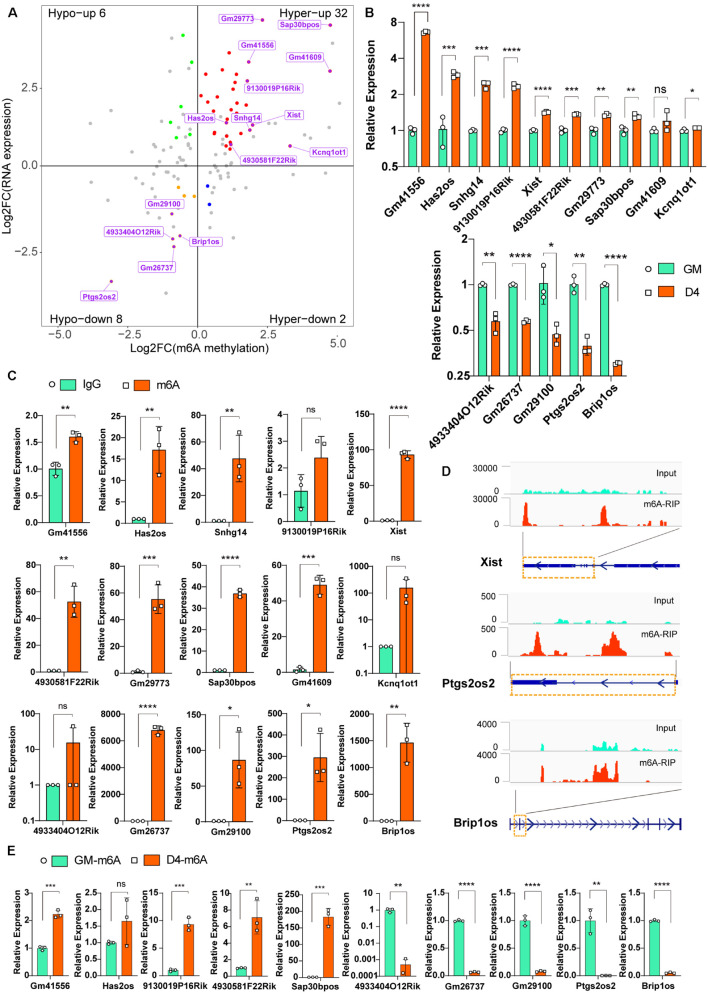
Differentially m^6^A modified lncRNAs in undifferentiated and differentiated muscle. **(A)** Distribution of genes with a significant change in both the m^6^A methylation and RNA expression levels before (GM) and after differentiation (D4), different colors were used to identify representative genes. And 15 m^6^A methylated significantly differently expressed lncRNAs were marked. **(B)** qRT-PCR validated the 15 m^6^A methylated significantly differently expressed lncRNAs in developing muscle cells. **(C)** Real-time PCR detection of the expression of the 15 m^6^A methylated significantly differently expressed lncRNAs in immunoprecipitated RNAs. IgG Immunoprecipitation was used as negative control. Quantitative data was represented as Mean ± SD; *p* value: *p < 0.05, **p < 0.01, ***p < 0.001, ****p < 0.0001. ns, no significant difference. **(D)** Integrative Genomics Viewer (IGV) plots show the m^6^A peaks of lncRNA Xist, Ptgs2os2 and Brip1os were highly enriched in m^6^A-RIP data. **(E)** Real-time PCR detection of the expression of the 5 hyper-upregulated and 5 hypo-downregulated lncRNAs in immunoprecipitated RNAs of GM and D4. Data were normalized by IgG Immunoprecipitation. Quantitative data was represented as Mean ± SD; *p* value: *p < 0.05, ** *p* < 0.01, ***p < 0.001, ****p < 0.0001. ns, no significant difference.

To explore the putative function of m^6^A on lncRNAs, we investigated the correlation between lncRNA transcript abundance and m^6^A methylation. In total, 123 significantly differentially m^6^A-methylated lncRNAs were expressed during myogenesis, as shown by RNA-seq. Among these lncRNAs, we identified 34 hypermodified lncRNAs (32 hyper-upregulated and 2 hyper-downregulated) and 14 hypomodified lncRNAs (6 hypo-upregulated and 8 hypo-downregulated) according to the criteria of adjusted *P* ≤ 0.05 and FDR ≤ 0.05 (Figure 3A). These results indicate a temporal difference in m^6^A methylation in differentially expressed lncRNAs. To verify the relationship between m^6^A level changes of lncRNAs and expression changes of lncRNAs, we randomly selected 15 significantly differentially expressed lncRNAs (Table 2) and performed qRT-PCR analysis. The results showed that 10 hyper-upregulated lncRNAs increased and 5 hypo-downregulated lncRNAs decreased in D4 (Figure 3B). These findings were consistent with sequencing data. It is worth mentioning that lncRNA Brip1os, which is significantly downregulated in D4 samples, as shown in Figure 1H, was accompanied by a decline in m^6^A modification.

**TABLE 2:** Randomly selected 15 altered m^6^A peaks in myoblasts and differentiated myotube.

Chr	Peak_start	Peak_end	Strand	Ensembl_ID	Gene_Name	RNA-seq_log2FoldChange	RNA-seq_padj	MeRIP-seq_diff.log2.fc	MeRIP-seq_diff.lg.fdr	Gene_Type	Group
11	115946568	115946986	–	ENSMUSG00000087064	Sap30bpos	4.068002111	0.000201133	4.75	−1.71	antisense	Hyper-up
17	70800582	70800823	–	ENSMUSG00000117231	Gm41609	2.740191127	0.003528931	4.74	−1.37	processed_transcript	Hyper-up
7	143257440	143257921	–	ENSMUSG00000101609	Kcnq1ot1	0.575398413	0.013352397	3.31	−1.51	antisense	Hyper-up
8	123899672	123900620	+	ENSMUSG00000110547	Gm29773	4.214800444	4.74E-15	2.32	−3.89	antisense	Hyper-up
X	103469670	103469796	–	ENSMUSG00000086503	Xist	1.181915678	6.27E-20	1.97	−49	lincRNA	Hyper-up
7	59970257	59973787	–	ENSMUSG00000100826	Snhg14	1.035494247	0.000971565	1.87	−6.16	processed_transcript	Hyper-up
17	29059068	29059722	+	ENSMUSG00000117007	Gm41556	3.003943093	0.001225038	1.83	−1.4	lincRNA	Hyper-up
6	54269710	54270309	–	ENSMUSG00000073067	9130019P16Rik	2.455499081	2.29E-06	1.78	−1.7	processed_transcript	Hyper-up
9	35126228	35126888	+	ENSMUSG00000070315	4930581F22Rik	0.617762934	0.032115595	1.19	−5.53	processed_transcript	Hyper-up
15	56694041	56765501	+	ENSMUSG00000086541	Has2os	1.24715012	0.012876036	1.03	−6.52	processed_transcript	Hyper-up
1	150159072	150159782	–	ENSMUSG00000097754	Ptgs2os2	−3.338667012	1.71E-06	−3.09	−2.46	lincRNA	Hypo-down
1	91801436	91801917	–	ENSMUSG00000100980	Gm29100	−1.386873948	0.044030192	−0.922	−1.41	antisense	Hypo-down
5	136933475	136934126	+	ENSMUSG00000097908	4933404O12Rik	−2.110740583	6.83E-22	−0.891	−1.35	lincRNA	Hypo-down
9	44112922	44113191	+	ENSMUSG00000097467	Gm26737	−2.335487486	1.12E-07	−0.849	−1.99	antisense	Hypo-down
11	86209134	86209584	+	ENSMUSG00000085208	Brip1os	−2.020791677	2.06E-50	−0.631	−3.96	antisense	Hypo-down

To verify the significantly differentially m^6^A-modified lncRNAs, we used an antibody against m^6^A and performed RNA immunoprecipitation followed by real-time PCR (m^6^A-IP-qPCR). As shown in Figure 3C, compared to that in the IgG control, most of the lncRNAs in Figure 3B were significantly enriched in the m^6^A group, indicating that these transcripts were m^6^A enriched. For example, the enrichment of Xist, Ptgs2os2 and Brip1os was elevated up to hundreds of thousands of fold in the m^6^A group, which is consistent with the MeRIP-seq data that revealed clear m^6^A peaks around their RNAs (Figure 3D). Furthermore, we verified the m^6^A peaks enrichment of 5 hyper-upregulated and 5 hypo-downregulated lncRNAs in GM and D4. By normalization of each group of Normal IgG, the m^6^A-IP-qPCR data was consistent with Figure 3A (Figure 3E). In summary, these results demonstrated that m^6^A methylation in lncRNAs is involved in myogenesis.

### m^6^A Methylation Levels Were Positively Correlated With the Abundance of lncRNAs

**FIGURE 4:**
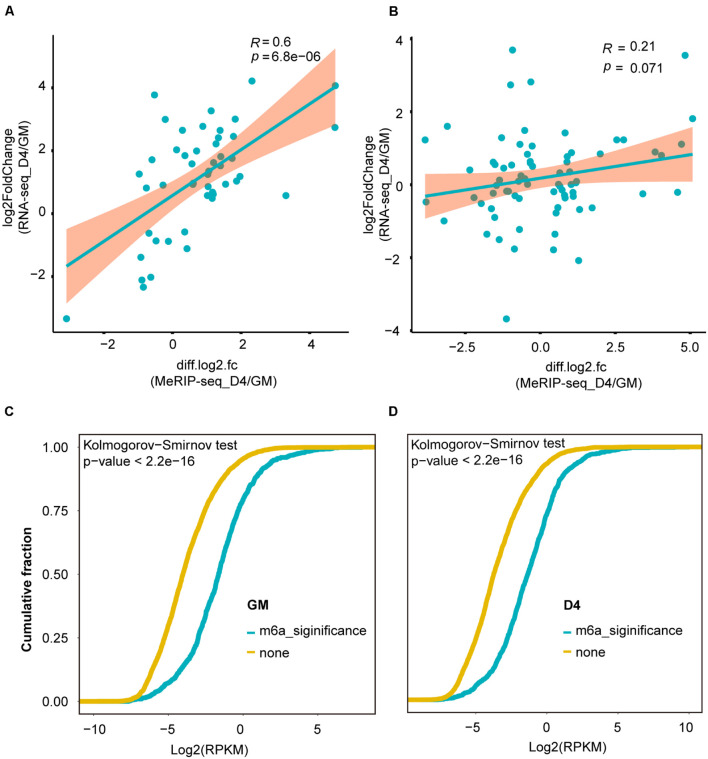
m^6^A methylation levels were positively correlated with the abundance of lncRNAs. **(A)** Scatter plot shows the positive correlation between m^6^A levels (significant changes) and expression values of lncRNAs with significantly differential expression between GM and D4, adjusted *P* ≤ 0.05. **(B)** Scatter plot shows no correlation between m^6^A levels (no significant changes) and expression values of lncRNAs without significantly differential expression between GM and D4. **(C)** Cumulative frequency of log2FC for lncRNAs containing m^6^A or without m^6^A methylation in GM. Kolmogorov-Smirnov test was used to estimated inter-group difference. **(D)** Cumulative frequency of log2FC for lncRNAs containing m^6^A or without m^6^A methylation in D4. Kolmogorov-Smirnov test was used to estimated inter-group difference.

More recently, m^6^A modification of mRNA was established to influence RNA stability dynamics and translation efficiency, and rapidly accumulating evidence shows significant crosstalk between lncRNA methylation and m^6^A-mediated epigenetic mechanisms (Kan et al., 2021). We then examined the correlation of lncRNA expression abundance with m^6^A methylation levels. For lncRNAs with significant expression abundance changes, their m^6^A levels were positively correlated with their expression levels (R = 0.6, *P* = 6.8e-6) (Figure 4A). However, for lncRNAs without significant transcript abundance changes, no significant correlation was noted between their m^6^A levels and expression levels (R = 0.21, *p* = 0.071) (Figure 4B). Interestingly, in both GM and D4 samples, lncRNAs with m^6^A methylation showed higher expression levels than those without m^6^A methylation (Figures 4C,D). Our analysis reveals that m^6^A methylation levels exhibit a positive correlation with the expression levels of m^6^A-modified lncRNAs and highlights the importance of m^6^A methylation in myogenesis-related lncRNAs.

### Myogenesis-Related lncRNAs Are Regulated by the m^6^A Methyltransferase METTL3

**FIGURE 5:**
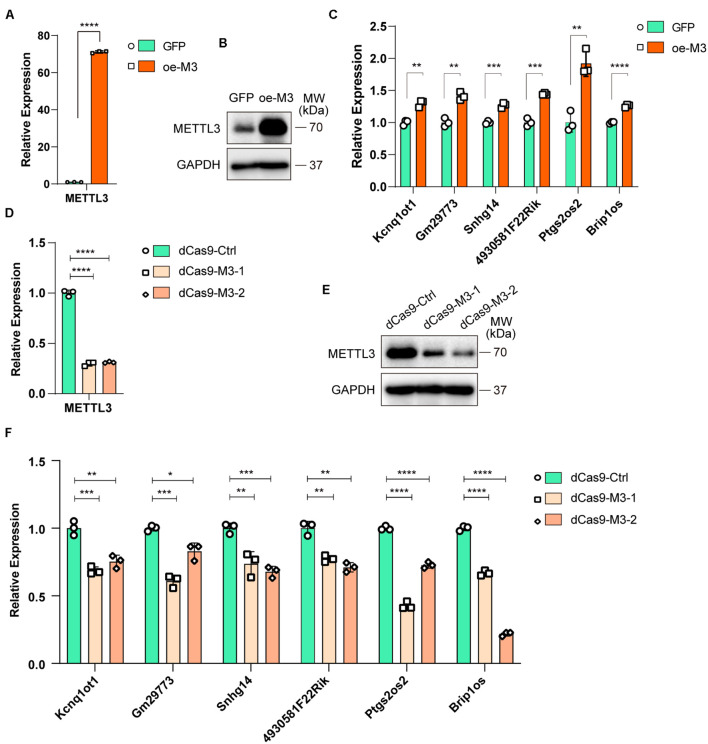
Myogenesis associated lncRNAs are regulated by m^6^A methyltransferase METTL3. **(A)** qRT-PCR shows the RNA expression level of METTL3 in METTL3-overexpressing C2C12 cells. GFP-overexpressing C2C12 cells as negative control. **(B)** Western blot detected the protein expression levels of METTL3 in METTL3-overexpressing C2C12 cells. **(C)** qRT-PCR shows the expression of myogenesis associated lncRNAs in METTL3-overexpressing C2C12 cells. **(D)** qRT-PCR shows the RNA expression level of METTL3 in METTL3 knockdown C2C12 stable cell lines. A nonsense sequence constructed to dCas9 repressor as negative control. **(E)** Western blot detected the protein expression levels of METTL3 in METTL3 knockdown C2C12 stable cell lines. **(F)** qRT-PCR shows the expression of myogenesis associated lncRNAs when METTL3 was knockdown. Data are presented as Mean ± SD; *p* value: *p < 0.05, **p < 0.01, ***p < 0.001, ****p < 0.0001.

To further investigate whether altered m^6^A modification levels could affect lncRNA expression, we overexpressed METTL3 in C2C12 cells (oe-M3), and GFP-overexpressing cells were used as a negative control (GFP). The real-time PCR and Western blot results validated that METTL3 was successfully overexpressed (Figures 5A,B). Then, we assayed the expression levels of six selected lncRNAs in GFP- and METTL3-overexpressing cells. As shown in Figure 5C, all 6 lncRNAs, including Brip1os, were significantly upregulated when METTL3 was overexpressed.

To validate the METTL3 overexpression results, we designed two gRNAs downstream of transcription start sites and used them to guide the fusion of inactive Cas9 (dCas9) to the Krüppel-associated box (KRAB) repressor to inhibit the transcription of METTL3 in C2C12 cells. METTL3 mRNA and protein levels were greatly reduced compared to those with control gRNA (Figures 5D,E). Then, we assayed the 6 lncRNA expression levels, and qPCR results showed that the expression levels of these lncRNAs decreased when METTL3 was knocked down (Figure 5F). Taken together, our results revealed that lncRNA expression is positively correlated with m^6^A modification levels during myogenesis, and it might be a universal regulation way that m^6^A modification levels affect lncRNAs abundance.

### m^6^A-Methylated lncRNAs Regulate Nearby mRNAs and Contribute to Muscle Tissue Development

**FIGURE 6:**
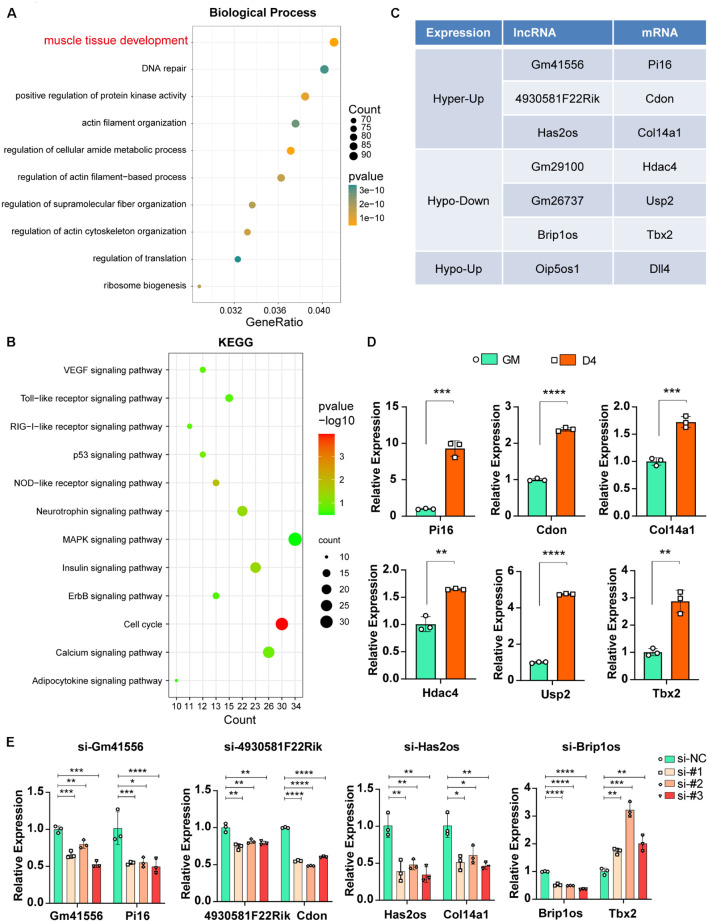
Functional relevance between m^6^A methylated lncRNAs and their adjacent mRNAs. **(A)** The top ten GO terms of the adjacent mRNAs (adjusted *P* ≤ 0.05) that related to differentially expressed lncRNAs in muscle cells. **(B)** The top twelve KEGG pathways of the adjacent mRNAs (adjusted *P* ≤ 0.05) that related to differentially expressed lncRNAs in muscle cells. **(C)** Table shows seven pairs of significantly differently expressed and methylated lncRNAs and their adjacent mRNAs in muscle cells. All have a significant threshold of FDR-adjusted *p* value ≤0.05. FDR, False Discovery Rate. **(D)** qRT-PCR shows the adjacent mRNA expression in GM and D4. **(E)** The effects of si-lncRNAs on the RNA expression levels of the corresponding lncRNA and mRNA. Data are presented as Mean ± SD; *p* value: *p < 0.05, **p < 0.01, ***p < 0.001, ****p < 0.0001.

Previous studies have reported that lncRNAs function in various physiological and pathological processes by regulating their adjacent mRNAs, either positively or negatively (Engreitz et al., 2016). Thus, we analyzed the significantly differentially expressed lncRNAs (FDR ≤ 0.05) during myogenesis as well as their nearest 10 mRNAs (upstream and downstream 5, respectively, adjusted *P* ≤ 0.05). GO analysis of biological processes for these mRNAs showed that 94 mRNAs were related to muscle tissue development (Figure 6A, adjusted *P* ≤ 0.05). KEGG pathway enrichment analysis showed that the cell cycle and MAPK signaling pathways were significantly enriched (Figure 6B, adjusted *P* ≤ 0.05), and such results are consistent with our previous studies showing that the JNK/MAPK and P38/MAPK signaling pathways play essential roles in myogenesis (Xie et al., 2018; Liu et al., 2021).

To confirm that m^6^A-methylated lncRNAs could regulate their adjacent mRNAs, we performed a synthetic analysis of 94 muscle tissue development-related mRNAs and nearby m^6^A-methylated lncRNAs. Briefly, given that nearby lncRNAs of these 94 mRNAs have significantly different expression, we further estimated m^6^A difference of these lncRNAs between GM and D4. As a result, seven lncRNAs have a significant m^6^A difference, and these lncRNAs also correspond to seven mRNAs (Figure 6C). Furthermore, mRNA expression of these paired adjacent mRNAs was tested, and qPCR results showed that Pi16, Cdon and Col14a1 were upregulated in D4 samples, consistent with their adjacent lncRNAs Gm41556, 4930581F22Rik, and Has2os, respectively. In contrast, Hdac4, Usp2 and Tbx2 were upregulated, which is an opposite effect compared with that noted in their adjacent lncRNAs (Figure 6D). These data indicated two opposite regulatory mechanisms between m^6^A-methylated lncRNAs and their nearby mRNAs, including positive and retrograde regulation.

Next, we further confirmed the regulation between lncRNAs and their adjacent mRNAs by knocking down corresponding lncRNAs using siRNAs. As shown in Figure 6E, when lncRNAs Gm41556, 4930581F22Rik, and Has2os were knocked down, their adjacent mRNAs Pi16, Cdon and Col14a1 were downregulated correspondingly, verifying the positive regulation between these lncRNAs and their nearby mRNAs. In contrast, when lncRNA Brip1os was knocked down, its adjacent mRNA Tbx2 was significantly upregulated, suggesting negative regulation. Taken together, our results showed that knockdown of m^6^A-methylated lncRNAs impacted the expression of their adjacent mRNAs and suggested that the functional relevance of m^6^A-methylated lncRNAs by regulating their adjacent mRNAs.

### The METTL3/m^6^A/Brip1os/Tbx2 Axis in Muscle Development

**FIGURE 7:**
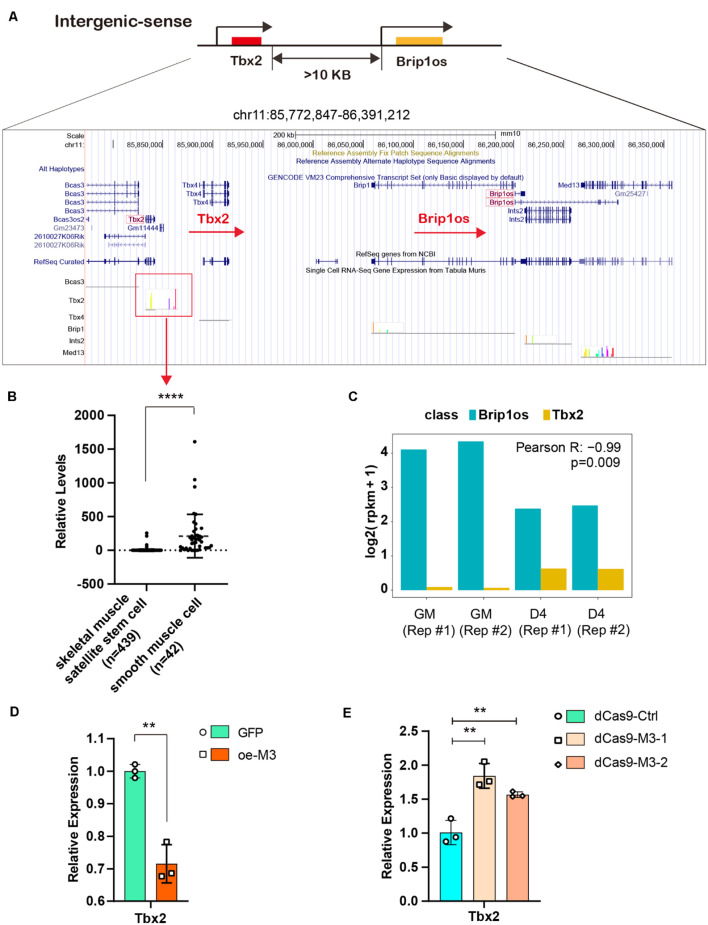
METTL3/m^6^A/Brip1os/Tbx2 axis in muscle development. **(A)** The genomic location of lncRNA Brip1os and its adjacent mRNA Tbx2. **(B)** Tbx2 expression data from public record of single cell RNA-seq gene expression of Tabula Muris data. **(C)** Brip1os and Tbx2 expression levels in our RNA-seq data. Pearson correlation analysis was performed to estimate expression correlation between two genes. **(D)** qRT-PCR shows the expression of Tbx2 when METTL3 overexpressed. **(E)** qRT-PCR shows the expression of Tbx2 when METTL3 was knockdown. Data are presented as Mean ± SD; *p* value: **p < 0.01, ****p < 0.0001.

Given that the lncRNA Brip1os exhibits markedly decreased expression and m^6^A modification levels during myogenesis and a perfectly negative correlation is noted between Brip1os and its nearby gene Tbx2, we further clarified their relationship. Brip1os and Tbx2 are both located at chr11qC, and these two transcription units have the same orientation. Brip1os is greater than 10 kb downstream of Tbx2 (359,435 bp) in the genome (Figure 7A). We further examined the expression of Tbx2 in muscle development by using single-cell RNA-seq data from Tabula Muris^1^ and analyzed the expression levels in skeletal muscle satellite stem cell and smooth muscle cell groups, which could be considered generally representative of undifferentiated myoblasts and differentiated myotubes, respectively. Surprisingly, although there were 439 samples in the skeletal muscle satellite stem cell group and only 42 samples in the smooth muscle cell group, Tbx2 expression levels in the smooth muscle cell group were significantly greater than those in the skeletal muscle satellite stem cell group (Figure 7B). Such results implied that Tbx2 was upregulated during skeletal muscle development, which was consistent with our qPCR results.

As shown in Figure 7C, Brip1os was highly expressed in two GM samples and decreased in two D4 samples, in which Tbx2 exhibited the opposite trend (Pearson R = −0.99, p = 0.009). These results confirm their negative correlation with RNA expression. Next, we assessed whether METTL3 affects Tbx2 expression. As shown in Figure 7D, the Tbx2 mRNA levels were greatly downregulated when METTL3 was overexpressed. Accordingly, Tbx2 mRNA levels were upregulated when METTL3 was knocked down (Figure 7E). These results suggest that Tbx2 could be regulated by METTL3. Taken together, we validated the retrograde regulatory relationship between Brip1os and Tbx2 as well as their responsive reaction to changes in the m^6^A methyltransferase METTL3.

## Discussion

Skeletal muscle development is precisely regulated in a sophisticated spatiotemporal manner. Our previously identified changes in gene expression and epigenetic modifications during skeletal muscle development have greatly improved our understanding of the mechanism related to myogenesis, including coding gene and non-coding RNA modifications (Diao et al., 2021a, b), mRNA expression (Tan et al., 2021), miRNA regulation (Xie et al., 2013, 2018), and lncRNA function (Liu et al., 2021). Taking advantage of the sequencing approach and gene annotation, a significant number of lncRNAs have been shown to play crucial roles in skeletal muscle development (Luo et al., 2021). Given the robust function of m^6^A methylation, the functions of m^6^A-modified mRNAs in the process of skeletal muscle development have been well studied, and the role of m^6^A-modified non-coding RNAs has also been appreciated (Li et al., 2021). In the present study, we hypothesized that lncRNAs might also be modified by m^6^A and participate in skeletal muscle differentiation. In the current study, we provided the first evidence that both the lncRNA transcriptome and m^6^A epitranscriptome underwent highly dynamic changes throughout mouse skeletal muscle development. Such results are consistent with results from other studies investigating the role of m^6^A-lncRNA in tissue development, such as those for mouse embryonic stem cell differentiation (Wang et al., 2014). Specifically, we observed that m^6^A methylation levels of lncRNAs are positively correlated with the transcript abundance of lncRNAs. Moreover, our studies revealed that lncRNAs exhibit pairwise expression correlations with neighboring mRNAs. Our results highlight a potential role of m^6^A-modified lncRNAs during skeletal muscle development.

LncRNAs are more cell-specific than other RNAs, and their expression models are not completely understood. Given that m^6^A is a ubiquitous modification in RNAs and regulates gene expression, we systematically identified m^6^A-modified lncRNAs and uncovered the m^6^A marks affecting the expression of lncRNAs. Our data showed that for lncRNAs with significant expression abundance changes, their m^6^A levels were positively correlated with their expression levels. However, for lncRNAs without significant transcript abundance changes, no significant correlation was noted between their m^6^A levels and expression levels. These results were further verified by overexpression or knockdown of the m^6^A core methyltransferase METTL3.

Due to the poor conservation of lncRNAs, their function and regulatory mechanisms are not completely understood. It is known that lncRNAs can regulate the expression of neighboring genes by cis-acting mechanisms (Lee, 2012). Mancini-DiNardo et al. clarified that elongation of the Kcnq1ot1 transcript is required for genomic imprinting of neighboring genes (Mancini-DiNardo et al., 2006). Furthermore, Ponjavic et al. noted that spatiotemporal coexpression of ncRNAs and nearby protein-coding genes represents a general phenomenon and presented substantive and predictive criteria for prioritizing lncRNA and mRNA transcript pairs when investigating their biological functions (Ponjavic et al., 2009). This information led us to hypothesize that m^6^A-methylated lncRNAs regulate nearby mRNAs and contribute to muscle tissue development. This hypothesis was supported by our computational analysis and experimental results. In particular, Brip1os is the most significantly differentially expressed and m^6^A-modified lncRNA, and its nearby mRNA Tbx2 plays an important function in muscle tissue development. The change in Tbx2 mRNA expression was opposite to that of Brip1os in D4 compared to GM. These findings indicate that they exhibit a retrograde regulatory relationship, which was verified using a public dataset. In addition, it has been known for decades that Tbx (T-Box) genes play crucial roles in limb development (Zhu et al., 2014; Pflugfelder et al., 2017). Further studies on their regulation are warranted. Our analysis provided candidate lncRNAs and mRNAs for further examination of the gene regulation network in muscle development.

The transition from myoblast proliferation to differentiation is accompanied by drastic alterations in the transcriptome. Transcriptional changes influencing muscle status are affected by a number of processes involving DNA, RNA and proteins. This study uncovered the differential expression and m^6^A methylation status of lncRNAs with temporal-specific expression in developing muscle. Surprisingly, we found no differences in the methylation of lncRNAs that drive myoblast state changes, such as linc-MD1 (Cesana et al., 2011) or lncMyoD (Dong et al., 2020), suggesting that these lncRNAs are not direct targets of dynamic m^6^A modification. Kcnq1ot1, a differentially expressed and m^6^A-enriched lncRNA identified in our study, participates in the regulation of genes within the Kcnq1 imprinting domain (Zhang et al., 2014) and controls maternal p57 expression in muscle cells by promoting H3K27me3 accumulation in an intragenic MyoD-binding region (Andresini et al., 2019). Intriguingly, of the top m^6^A-enriched lncRNA transcripts in GM and in D4, many have not been previously linked to myoblast/myotube function but have been reported to be involved in other cell types. For example, Snhg14 (small nucleolar RNA host gene 14) is expressed at elevated levels among the top ten differentially m^6^A-enriched lncRNAs in D4 compared to GM. Snhg14 is highly expressed in Parkinson’s disease, and silencing Sngh14 mitigated dopaminergic neuron injury by downregulating a-syn by targeting miR-133b (Zhang et al., 2019). In addition, Sngh14 functions as a ceRNA in Ang II-induced cardiomyocytes to sponge both miR-322-5p and miR-384-5p to elevate PCDH17 levels (Long et al., 2020). The function of m^6^A-modified lncRNAs during skeletal muscle development needs to be further studied.

In summary, we described the expression and m^6^A methylation profiles of lncRNAs that display temporal expression in mouse myoblasts and differentiated myotubes. Our findings provide new insight into the pivotal regulatory role of m^6^A-modified lncRNAs in muscle development. Our data uncovered the novel posttranscriptional regulation underlying muscle differentiation and provide a molecular basis for further studies to determine the function and mechanism of m^6^A-lncRNAs in skeletal muscle development and muscle-related diseases.

## Data Availability Statement

The datasets presented in this study can be found in online repositories. The names of the repository/repositories and accession number(s) can be found in the article/Supplementary Material.

## Ethics Statement

The animal study was reviewed and approved by the Institutional Animal Care and Use Committee of Sun Yat-sen University.

## Author Contributions

S-JX, Z-DX, QZ, and Y-WP conceived and designed this project. S-JX, ST, and L-TD performed the experiments with the help of Y-XH, Y-RH, HL and, W-YX. S-JX, W-JC, Z-GZ, P-LL, and W-CC analyzed the data. S-JX, Z-DX, ST, L-TD, Y-WP, and QZ wrote the manuscript. All authors contributed to the article and approved the submitted version.

## Conflict of Interest

The authors declare that the research was conducted in the absence of any commercial or financial relationships that could be construed as a potential conflict of interest.

## Publisher’s Note

All claims expressed in this article are solely those of the authors and do not necessarily represent those of their affiliated organizations, or those of the publisher, the editors and the reviewers. Any product that may be evaluated in this article, or claim that may be made by its manufacturer, is not guaranteed or endorsed by the publisher.

## Footnotes

1
http://genome.ucsc.edu
