# Supplementary figures and images for: Characterization of Long Non-coding RNAs Modified by m6A RNA Methylation in Skeletal Myogenesis
Source: Front Cell Dev Biol. 2021 Oct 13;9:762669. doi: 10.3389/fcell.2021.762669 (PMC8548731; doi:10.3389/fcell.2021.762669)

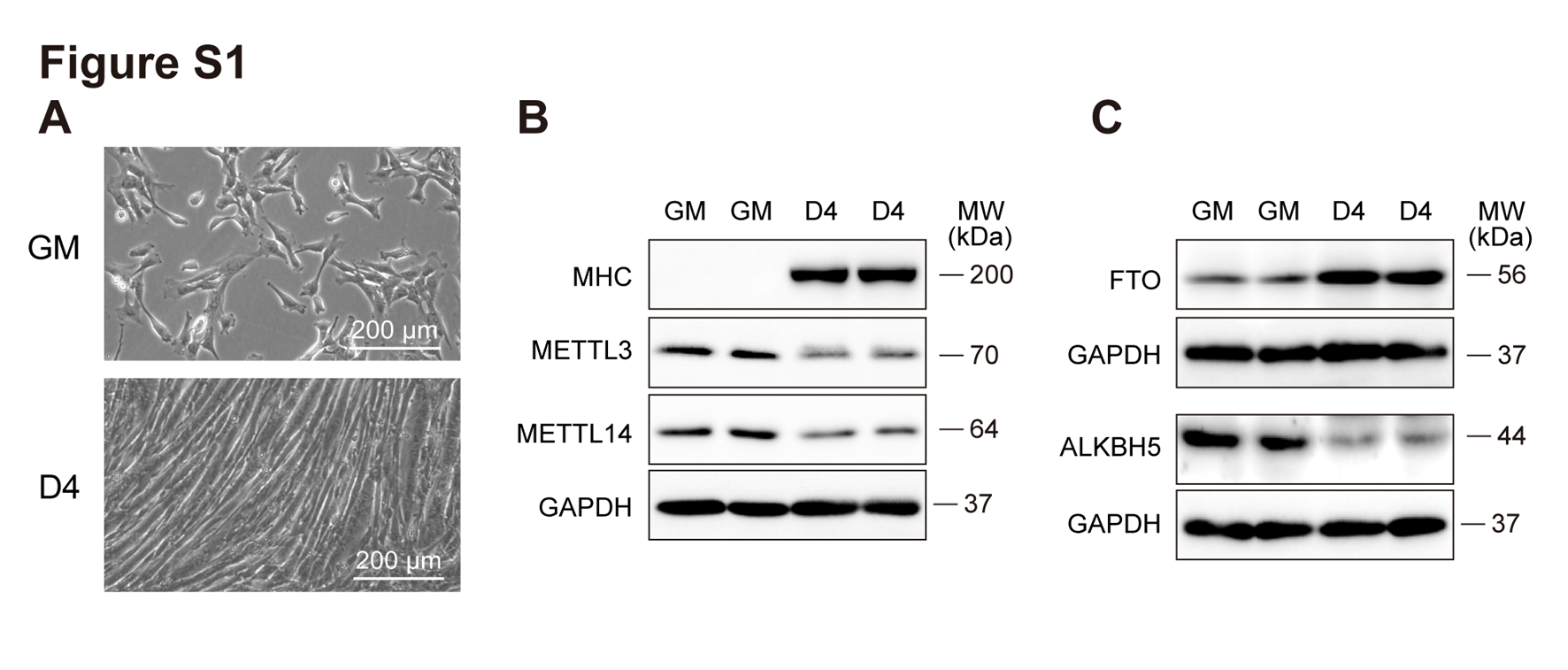

Supplement: Supplementary file 1 [file Image_1.TIF]

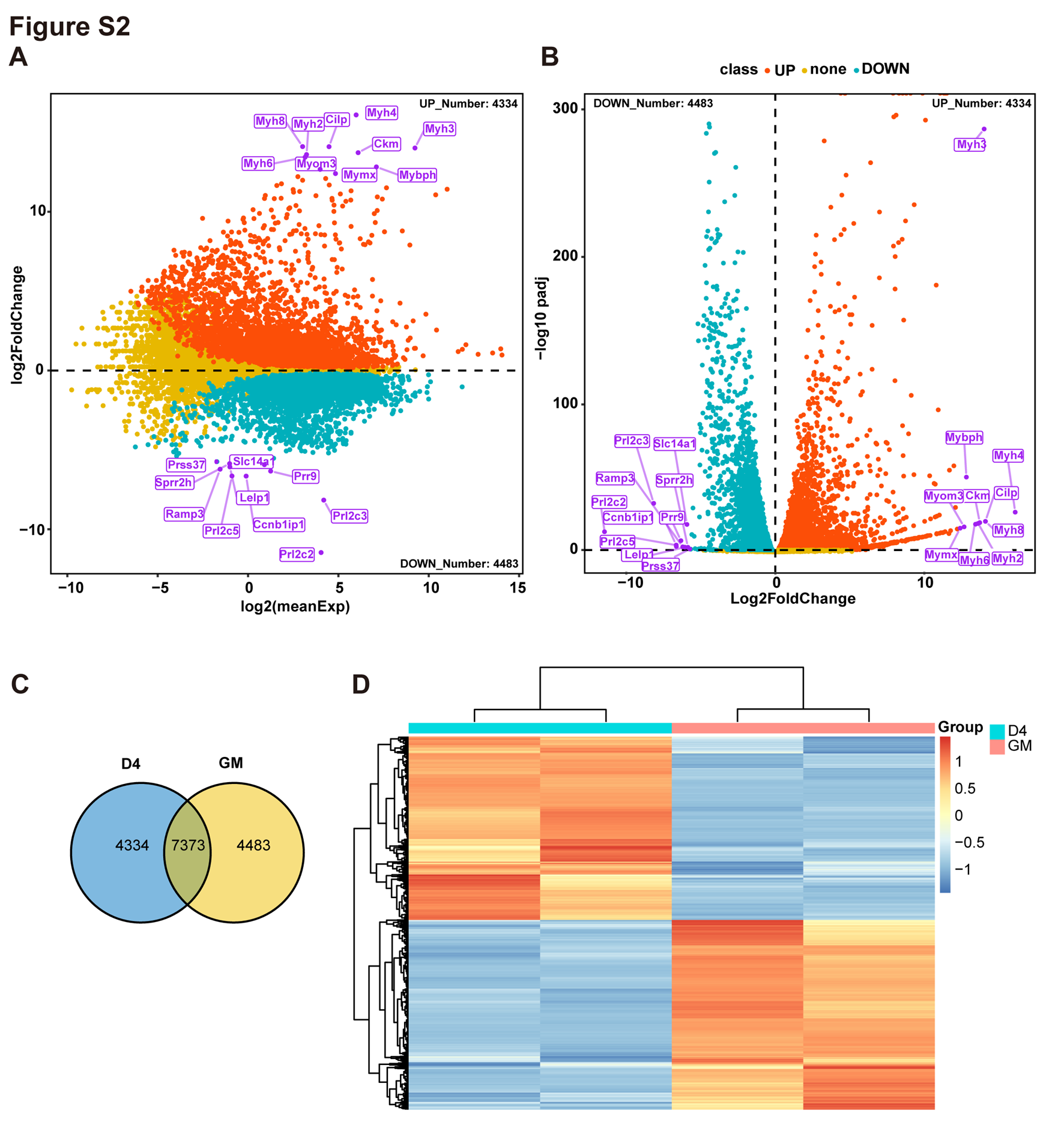

Supplement: Supplementary file 2 [file Image_2.TIF]

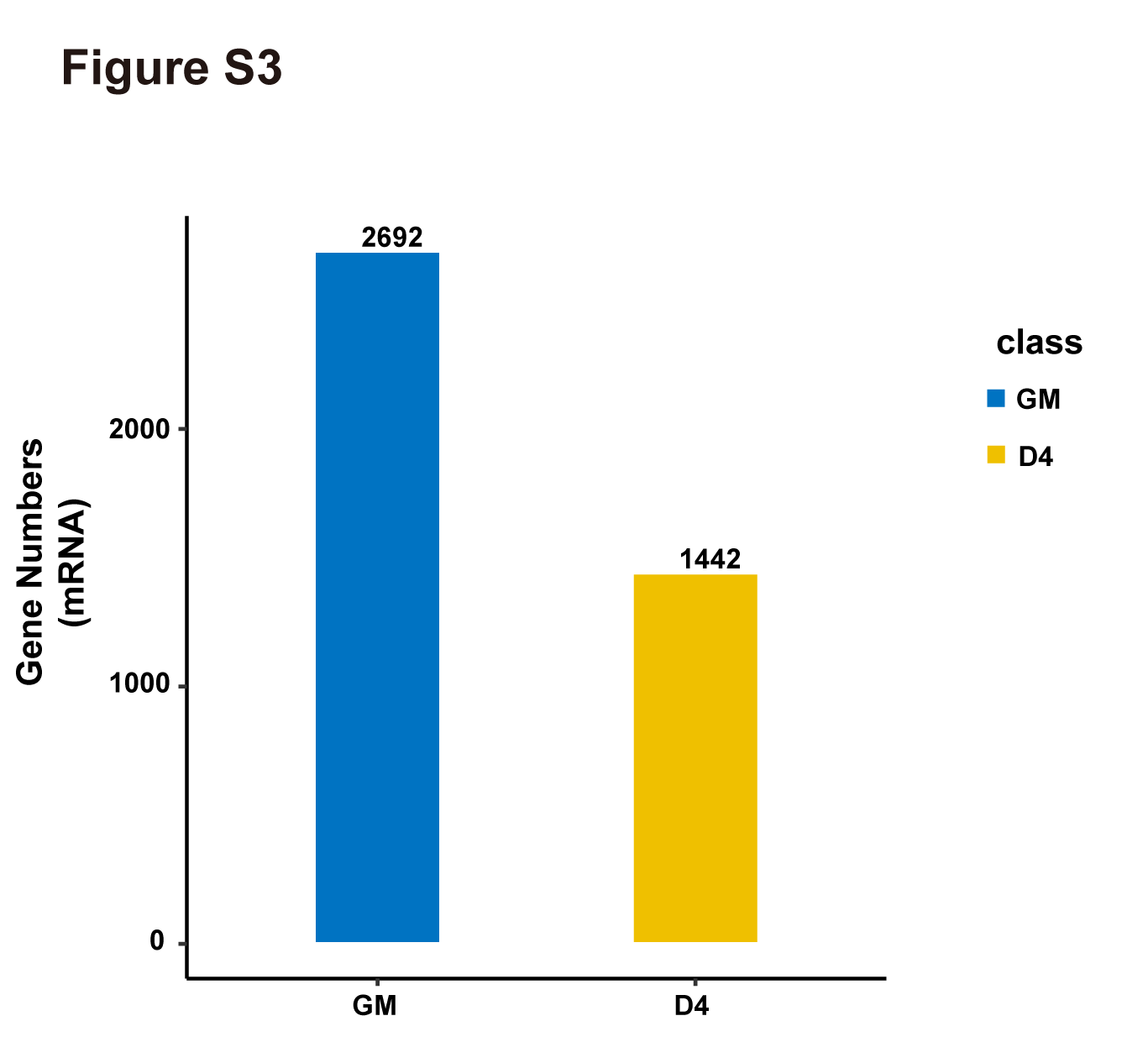

Supplement: Supplementary file 3 [file Image_3.TIF]
