# Supplementary material for: Characterization of Long Non-coding RNAs Modified by m6A RNA Methylation in Skeletal Myogenesis
Source: Front Cell Dev Biol. 2021 Oct 13;9:762669. doi: 10.3389/fcell.2021.762669 (PMC8548731; doi:10.3389/fcell.2021.762669)
Supplement: Supplementary file 4 [file Data_Sheet_1.docx]

**Supplementary Information**

Attached to Manuscript

Xie *et al*. “Characterization of long noncoding RNAs modified by m6A RNA methylation in skeletal myogenesis”

**Titles and Legends to Supplementary Figures**

**Supplementary Figure 1. METTL3/14 expression levels decline during myoblast differentiation.**

1. Morphology of C2C12 myoblasts in growth medium (GM), and in differential medium for 4 days (D4) after reaching a confluence about 90%.
2. Western blot analysis of core components of m^6^A methyltransferases METTL3 and METTL14 of C2C12 myoblasts in GM and D4.
3. Western blot analysis of m^6^A demethylases FTO and ALKBH5 of C2C12 myoblasts in GM and D4.

**Supplementary Figure 2. Dynamic profile of mRNAs in myoblasts and differentiated myotubes.**

1. MA plot shows the relationship between expression abundance and fold changes of mRNAs in myoblasts (GM) and myotube (D4). log2 (MeanExp) represents gene expression values, log2Fold Change represents the fold change of mRNAs at D4 compared to GM. Red dots represent 4334 significantly up-regulated mRNAs at D4 in relation to GM, adjusted P ≤ 0.05; Green dots, represent 4483 significantly down-regulated mRNAs at D4 in relation to GM, adjusted P ≤ 0.05; Yellow dots represent mRNA without significantly differential expression, adjusted P > 0.05. Top differentially expressed mRNAs were marked in purple.
2. The volcano plot shows significantly differentially expressed mRNAs at GM and D4. Red dots represent 4334 significantly up-regulated mRNAs at D4, adjusted P ≤ 0.05; Green dots, represent 4483 significantly down-regulated mRNAs at D4, adjusted P ≤ 0.05; Yellow dots represent mRNA without significantly differential expression, adjusted P > 0.05. Top differentially expressed mRNAs were marked in purple.
3. The Venn diagram shows 4483 and 4334 significantly differentially expressed mRNAs in GM and D4, and 7373 mRNAs without significant difference between GM and D4.
4. The heatmap shows significantly differentially expressed mRNAs in GM and D4. Orange color represents up-regulation in D4, while blue color represents down-regulation. Row represents genes, column represents samples, and each cell represents expression value.

**Supplementary Figure 3. Numbers of m^6^A methylated mRNA in undifferentiated and differentiated muscle.**

Bar plot shows the Numbers of m^6^A methylated mRNAs in GM and D4. Blue represents 2692 hyper-methylated mRNAs in GM, while yellow represents 1442 hyper-methylated mRNAs in D4.

**Titles to Supplementary Tables**

**Supplementary Table 1. The sequences of primers used in this study.**

**Supplementary Table 2.** **The sequences of siRNAs used in this study.**

**Supplementary Table 3.** lncRNA reads of RNA-seq sequencing data of C2C12 myoblasts in GM and D4.

**Supplementary Table 4.** mRNA reads of RNA-seq sequencing data of C2C12 myoblasts in GM and D4.

**Supplementary Table 5.** Unique m^6^A peaks of MeRIP-seq sequencing data of myoblasts (GM) (FDR ≤ 0.05).

**Supplementary Table 6.** Unique m^6^A peaks of MeRIP-seq sequencing data of myotubes (D4) (FDR ≤ 0.05).

**Supplementary Table 7.** lncRNA m^6^A peaks of MeRIP-seq sequencing data of myoblasts (GM) (FDR ≤ 0.05).

**Supplementary Table 8.** lncRNA m^6^A peaks of MeRIP-seq sequencing data of myotubes (D4) (FDR ≤ 0.05).

**Supplementary Table 9.** mRNA m^6^A peaks of MeRIP-seq sequencing data of C2C12 myoblasts in GM and D4 (FDR ≤ 0.05).
